# Nucleotide Diversity at Site 106 of *EPSPS* in *Lolium perenne* L. ssp. *multiflorum* from California Indicates Multiple Evolutionary Origins of Herbicide Resistance

**DOI:** 10.3389/fpls.2017.00777

**Published:** 2017-05-09

**Authors:** Elizabeth Karn, Marie Jasieniuk

**Affiliations:** Department of Plant Sciences, University of California, DavisDavis, CA, USA

**Keywords:** herbicide resistance, weed populations, *EPSPS* mutations, mechanism, evolutionary origins

## Abstract

The repeated evolution of herbicide resistance in weeds is an ongoing problem in agricultural regions across the world, and presents a unique system in which to study the origins and spread of adaptive traits across heterogeneous landscapes. *Lolium perenne* ssp. *multiflorum* (Lam.) (Italian ryegrass) is a widespread grass weed of agricultural crops that has repeatedly evolved resistance to herbicides across the world. In California, resistance to glyphosate has become increasingly common. To identify the mechanisms conferring glyphosate resistance in California populations of *L. perenne* and to gain insights into the evolutionary origins and spread of resistance in the region, we investigated the frequency of target-site mutations conferring resistance to glyphosate combined with the frequency of resistant individuals in 14 populations. A region of *5-enolpyruvylshikimate-3-phosphate synthase* (*EPSPS*) was sequenced in 401 individuals to assay for target site mutations. Seven unique alleles were detected at codon site 106, four of which have been previously shown to confer target-site-based resistance to glyphosate. Four different resistance alleles were detected, indicating that resistance to glyphosate has evolved multiple times in the region. In two populations, no *EPSPS* mutations were detected despite the presence of resistant plants, strongly suggesting that non-target-site-based mechanisms confer resistance to glyphosate in these populations. It is likely that resistance to glyphosate in these 14 California populations of *L. perenne* derives from at least five evolutionary origins, indicating that adaptive traits can evolve repeatedly over agricultural landscapes.

## Introduction

The phenomenon of agricultural weeds evolving in response to agricultural practices presents an ideal opportunity to study plant adaptation across landscapes. Agricultural landscapes are comprised of fields and the lands between them, and management by different growers or agencies across time and space results in a heterogeneous landscape of environments for weeds. Weed management practices such as tillage, hand weeding, and herbicide applications are strong selection pressures for the evolution of adaptive traits that allow plants to survive those management strategies (Barrett, 1983; Powles and Yu, 2010; Owen et al., 2011; Delye et al., 2013). In particular, repeated applications of herbicides with the same site of action selects for rare mutant resistant individuals which, if present, can result in a rapid increase in the frequency of resistance alleles and resistant plants in the population until the infestation becomes uncontrollable with those herbicides (Jasieniuk et al., 1996; Neve et al., 2009).

Repeated evolution of resistance to glyphosate, the most widely used herbicide worldwide (Baylis, 2000; Benbrook, 2016), has been particularly problematic, occurring in 36 weed species on six continents to date (Heap, 2016). Glyphosate inhibits 5-enolpyruvylshikimate-3-phosphate synthase (EPSPS), an enzyme in the shikimate pathway, which results in the accumulation of shikimate and plant death (Holländer and Amrhein, 1980; Steinrücken and Amrhein, 1980; Herrmann and Weaver, 1999). Following the widespread adoption of glyphosate-resistant transgenic crops and the associated increases in glyphosate use, many weed populations evolved multiple mechanisms of resistance to glyphosate (Baylis, 2000; Powles, 2008; Heap, 2016).

Non-synonymous mutations resulting in four different amino acid substitutions at codon site 106 of *EPSPS* have been shown to confer resistance to glyphosate in several weed species (Kaundun et al., 2011; Shaner et al., 2011). The mutations at site 106 result in an altered EPSPS enzyme that is not bound by glyphosate while retaining affinity for the PEP substrate, allowing plants to survive field-applied doses of the herbicide (Funke et al., 2006). All known mutations conferring target-site-based resistance occur at site 106 (Shaner et al., 2011). Although individuals with mutations at both sites 102 and 106 have been shown to confer a heightened level of resistance in goosegrass in Malaysia (Yu et al., 2015), non-synonymous mutations at site 102 alone do not confer resistance to glyphosate (Funke et al., 2006).

Resistance to glyphosate is also commonly conferred by altered translocation of glyphosate through the plant or by amplification of the *EPSPS* gene (Powles and Yu, 2010; Shaner et al., 2011). The first identified and most frequently cited mechanism of glyphosate resistance is altered translocation, where glyphosate is prevented from reaching its target site, the EPSPS enzyme, by translocation away from meristems and actively growing points and sequestration in the vacuole (Wakelin et al., 2004; Ge et al., 2010, 2012). The genetic basis of this mechanism of resistance to glyphosate is not currently known, although ATP-binding cassette (ABC) transporters and a tonoplast-intrinsic protein (TIP) have been implicated in glyphosate resistance in *Conyza canadensis* (Peng et al., 2010; Yuan et al., 2010). Recently, *EPSPS* gene amplification has been identified as a mechanism of resistance to glyphosate in multiple weed species (Sammons and Gaines, 2014). Resistant individuals with this mechanism may contain two to over 100 copies of *EPSPS* as a result of tandem gene duplication or a mobile genetic element, with correlative high *EPSPS* expression and resistance levels (Gaines et al., 2010; Jugulam et al., 2014; Wiersma et al., 2015).

*Lolium perenne* ssp. *multiflorum* is a diploid, self-incompatible, obligately outcrossing grass weed that infests a wide range of crops worldwide (Fearon et al., 1983; Charmet et al., 1996). Populations of *L. perenne* ssp. *multiflorum* and the closely related *Lolium perenne* ssp. *rigidum* have evolved resistance to herbicides with 12 different modes of action on six continents (Heap, 2016). In California, resistance to glyphosate in *L. perenne* ssp. *rigidum* was first identified in 1998 in an almond orchard (Simarmata et al., 2003), and later confirmed in multiple populations of *L. perenne* ssp. *multiflorum* across the Central Valley (Jasieniuk et al., 2008). Proline-to-alanine (P106A) or proline-to-serine (P106S) substitutions at the site corresponding to codon 106 of the *EPSPS* gene were identified in resistant plants (Jasieniuk et al., 2008; Simarmata and Penner, 2008). To date, non-target-site-based resistance to glyphosate has not been identified in California populations of *L. perenne* ssp. *multiflorum*. In 2013, populations of *L. perenne* ssp. *multiflorum* containing glyphosate-resistant plants were identified in northwestern California after 2 years of failed control with glyphosate. These populations are separated geographically from the Central Valley and may have evolved resistance independently through the same or a different mechanism.

While resistance to glyphosate has evolved repeatedly in multiple species and within species across different regions around the world (Heap, 2016), it is not always clear how the adaptive trait evolves and spreads among populations of a species within an agricultural region. Early population genetic models predicted that gene flow likely contributes to the spread of target-site-based resistance across a landscape to a greater degree than do novel mutations, as mutation rates are generally assumed to be low (Jasieniuk et al., 1996). Recently, studies of neutral genetic variation in weed populations, combined with patterns of phenotypic variation in resistance, have provided support for the spread of herbicide resistance through both gene flow (Delye et al., 2010a; Okada et al., 2013, 2014) and independent origins (Kuester et al., 2015). However, in weeds with highly outcrossing mating systems and high genetic diversity within populations, such as *L. perenne*, genetic differentiation between populations is often low (Balfourier et al., 1998; Kubik et al., 2001; McGrath et al., 2007; Wang et al., 2009), making it difficult to determine whether a trait shared by two populations is derived from a common origin.

Analysis of genetic diversity and population structure of California *L. perenne* ssp. *multiflorum* with microsatellite markers did not reveal whether the glyphosate resistance trait had originated once and then spread within and among populations, or spread as a result of multiple independent evolutionary origins (Karn and Jasieniuk, 2017). However, extensive population admixture did indicate the potential for resistance spread through gene flow. In this study, we examined genetic variation in *EPSPS* at codon site 106 where target-site mutations conferring resistance to glyphosate have previously been identified (Sammons and Gaines, 2014; Heap, 2016). If multiple alleles known to confer resistance are detected at this locus, then logically they must be derived from separate mutation events and, consequently, we can conclude that resistance has evolved multiple times. However, if all resistance alleles are identical, a single mutation event with subsequent gene flow through pollen or seed dispersal may have spread resistance among populations in the region. If resistance is observed in populations but resistant individuals do not contain any *EPSPS* mutations at codon site 106, resistance may be conferred by non-target-site-based mechanisms.

In addition to contributing basic knowledge on how adaptive traits originate and spread across landscapes, increased understanding of the evolution of herbicide resistance is needed to mitigate its impacts on agriculture (Neve et al., 2014). One possible strategy for the management of resistance with a single evolutionary origin relies on limiting the spread of resistance while using resistance management practices to control already resistant populations (Neve et al., 2009; Beckie, 2011). In contrast, when populations of a weed species have multiple independent origins of herbicide resistance, successful management requires the implementation of practices that reduce factors contributing to selection for resistance, in addition to limiting spread and controlling already resistant populations (Neve et al., 2009; Norsworthy et al., 2012).

The goal of this study was to examine the evolutionary origins of glyphosate resistance across a landscape by investigating *EPSPS* target site mutations in *L. perenne* ssp. *multiflorum* populations in northwest California. Specifically, we asked the following questions (i) which *EPSPS* alleles at site 106 are associated with glyphosate resistance in *L. perenne* populations in northwest California?, (ii) has resistance evolved more than once across the landscape?, and (iii) is there evidence that non-target-site-based resistance is present in California populations of *L. perenne*? To address these questions, we phenotyped individuals from multiple populations for resistance or susceptibility to glyphosate, sequenced the codon at *EPSPS* site 106 where target-site resistance mutations occur in both resistant and susceptible individuals, and assessed the frequency of different *EPSPS* alleles present in populations.

## Materials and Methods

### Plant Material and Glyphosate Resistance

Italian ryegrass populations in orchards and vineyards from Sonoma and Lake Counties where growers reported difficulty controlling plants with glyphosate, and from surrounding areas where predominantly susceptible populations may be experiencing gene flow with resistant plants, were sampled (**Table 1**). One additional population from Butte County in the Central Valley, from an area identified as containing evolved resistance to glyphosate more than 10 years ago (Simarmata et al., 2003; Jasieniuk et al., 2008), served as a comparison with the populations from Sonoma and Lake Counties where resistance has evolved more recently. In each of the populations, leaf tissue was collected for DNA extraction, and mature seeds were collected for resistance testing, from 30 to 40 randomly sampled individuals.

**Table 1:** *Lolium perenne* ssp. *multiflorum* populations sampled in northwest California and the numbers of genotyped and phenotyped individuals and frequencies of glyphosate-resistant plants in each.

Population ID	Cropping system	County	Latitude (N)	Longitude (W)	N_S_	N_G_	N_P_	%R
1	Orchard	Butte	39.8	-121.98	32	23	128	73.8
2	Vineyard	Sonoma	38.23	-122.52	30	27	128	9.7
3	Vineyard	Sonoma	38.24	-122.42	37	28	171	22.5
4	Vineyard	Sonoma	38.24	-122.36	33	18	212	29.1
6	Vineyard	Sonoma	38.359	-122.502	34	31	123	22.7
7	Vineyard	Sonoma	38.214	-122.457	33	32	65	32.1
8	Vineyard	Sonoma	38.587	-122.829	31	31	176	26.6
9	Vineyard	Sonoma	38.662	-122.825	33	29	150	31.6
10	Vineyard	Sonoma	38.673	-122.811	32	30	55	35.2
11	Vineyard	Sonoma	38.761	-122.976	41	39	166	40.6
12	Vineyard	Lake	38.989	-122.821	20	18	91	85.1
13	Orchard	Lake	38.997	-122.834	36	34	186	89
14	Orchard	Lake	38.996	-122.84	31	31	153	87.9
15	Orchard	Lake	39.086	-122.943	30	30	145	20.6


*N_S_, number of individuals sampled for leaf tissue and seeds from each population; N_G_, number of individuals genotyped; N_P_, number of progeny phenotyped for response to glyphosate; % R, percentage of individuals surviving treatment with glyphosate at 1678 g ae ha^-1^*.

To test for resistance to glyphosate, eight seeds from each sampled plant were germinated on moistened filter paper in Petri dishes at 20°C and a 12-h photoperiod. Germinated seedlings were transplanted into 8 cm × 8 cm square pots filled with UC soil mix (sand, compost, and peat in 1:1:1 ratio with 1.8 kg m^-3^ dolomite) with two seedlings per pot and grown in the greenhouse at 27/15°C with ambient light conditions. At the tillering stage, individual plants were divided into genetically identical clones following the method described by Boutsalis (2001) and grown in the greenhouse to the two to three leaf stage. One clone of a genotype was treated with water, which served as a control. The second clone was treated with glyphosate (Roundup PowerMax, Monsanto, St. Louis, MO, USA) at the rate of acid equivalent 1681 g ha^-1^, which is twice the recommended (label) field rate for the control of annual *L. perenne* plants under 6″ tall. All treatments were applied in an enclosed cabinet track sprayer equipped with an 8002E nozzle (TeeJet, Spraying Systems Co., Wheaton, IL, USA) delivering 200 L ha^-1^. Three weeks after glyphosate treatment, we scored each plant as alive or dead, and characterized the percentage of resistant plants in each population by the percentage of plants surviving glyphosate treatment of the total number of plants treated. Plants from a previously characterized susceptible reference seed collection (Jasieniuk et al., 2008) were included during each herbicide application to confirm herbicide activity.

### Detection of Target-Site Mutations

DNA was extracted from leaf tissue of all individuals sampled in the field following the CTAB method (Doyle and Doyle, 1987). Extracted DNA was quantified and diluted to 25 ng μL^-1^. The following primers used for PCR amplification of the region surrounding site 106 of *EPSPS* were designed from the *L. perenne* ssp. *multiflorum* GenBank sequence available at the time of genotyping (accessed on January 18, 2011): F: 5′-AACCGGATCCTCCTCCTCT-3′ and R: 5′-TGCCAAGGAAACAATCAACA-3′. *EPSPS* alleles were amplified in PCR reactions consisting of 25 ng DNA template, 1× Qiagen PCR buffer (Valencia, CA, USA), 0.25 mM additional MgCl_2_, 0.4 μM forward and reverse primers, 0.125 mM DNTPs, and 0.5 units Taq polymerase. The PCR program consisted of an initial denaturing period of 3 min at 94°C, followed by 30 cycles of 1 min at 94°C, 1 min at 57°C, 2 min at 72°C, and a final extension of 10 min at 72°C. PCR products were cleaned to remove excess nucleotides with ExoSAP-IT (Affymetrix, Santa Clara, CA, USA) solution according to the manufacturer’s instructions prior to amplification with BigDye Terminator v3.1 Cycle Sequencing Kit (Thermo Fisher Scientific, Waltham, MA, USA) following manufacturer instructions. Sequencing PCR products were precipitated in an ethanol and sodium acetate wash and resuspended in 10 μL highly deionized formamide.

Sequencing was performed with an ABI 3100 Genetic Analyzer (Applied Biosystems, Foster City, CA, USA). Sequences were edited with Sequencing Analysis Software v5.1 (Applied Biosystems), and aligned with Geneious Software (Auckland, New Zealand). Usable DNA sequences at site 106 were obtained from 401 individuals. The region corresponding to amino acid site 106, where point mutations conferring resistance to glyphosate were previously identified, was scanned for mutations. Individuals with single peaks at each base position were recorded as homozygous for that base, while individuals with multiple overlapping peaks at a base position were recorded as heterozygous for those two bases.

## Results

### Resistance to Glyphosate

Of the 1949 individuals tested for resistance to glyphosate at 1681 g ae ha^-1^, 38% survived herbicide treatment. All sampled populations contained some individuals that survived (**Table 1**). Within populations, resistance to glyphosate, estimated as the percentage of individuals surviving glyphosate treatment per population, varied from 9.7 to 89.0% (**Table 1**). Population 1, sampled from the area where glyphosate resistance was first reported in California (Simarmata et al., 2003), contained 73.8% resistant individuals. Three populations (populations 12, 13, and 14) from an area where growers reported possible resistance contained 85–89% resistant individuals, while a population (population 15) bordering the area contained 21% resistant individuals, confirming glyphosate resistance in the region. In the southern portion of the studied area, populations show a gradient of survivorship ranging from 9.7% survivorship in the southern end to 40.6% in the northern end of Sonoma County (**Table 1**).

### *EPSPS* Alleles

The region of the *EPSPS* gene encoding codon site 106 was sequenced from DNA of 401 field-sampled plants. Seven different alleles were identified at site 106 (**Table 2**). Three of these (CCA, CCC, and CCT) encode the wild-type susceptible proline allele (P106). The other four alleles contain non-synonymous mutations at site 106, which result in amino acid substitutions from proline to threonine (P106T), serine (P106S), leucine (P106L), and alanine (P106A). These four mutant resistance alleles were detected in 20.2% of all individuals genotyped and account for 11% of the 802 alleles detected across all populations.

**Table 2:** Numbers of different alleles and amino acid substitutions detected in 401 *Lolium perenne* ssp. *multiflorum* individuals genotyped at codon site 106 of *EPSPS*.

Population	CCA P106	CCC P106	CCT P106	ACA P106T	GCA P106A	TCA P106S	TTA P106L	Total detected
1	29	4	1	2	3	7	0	46
2	46	5	0	2	0	1	0	54
3	52	3	0	0	0	1	0	56
4	30	3	0	1	0	1	1	36
6	56	3	0	0	0	0	3	62
7	55	8	0	0	0	0	1	64
8	54	6	1	1	0	0	0	62
9	48	7	2	0	1	0	0	58
10	55	5	0	0	0	0	0	60
11	68	5	2	3	0	0	0	78
12	24	1	0	11	0	0	0	36
13	40	0	0	28	0	0	0	68
14	39	1	0	22	0	0	0	62
15	59	1	0	0	0	0	0	60

Total	655	52	6	70	4	10	5	802


*Two EPSPS alleles were detected per individual*.

The most common allele in all populations was P106 encoded by CCA, accounting for 655 of 802 alleles detected (**Table 2**), even in populations where the majority of individuals also contained a resistant allele. Most individuals containing a resistant allele were heterozygous, with one copy of a resistant allele and one copy of a susceptible allele (**Table 3**). Eight out of 81 individuals (10%) with resistance alleles were homozygous for the P106T allele, all of them in populations 13 or 14. No individuals were found to be heterozygous for two different resistance alleles, despite the presence of multiple types of resistance alleles in some populations.

**Table 3:** Frequencies of glyphosate-susceptible or -resistant allelic genotypes at site 106 and the frequencies of resistant phenotypes within sampled *Lolium perenne* ssp. *multiflorum* populations in northwest California.

	% S	% R				% RR	% R	Total % R	% R	
Population	P106	P106T	P106A	P106S	P106L	Alleles	Alleles	Alleles	Individuals	N_G_
1	47.8	8.7	13.0	30.4	0.0	0.0	52.2	52.2	73.8	23
2	88.9	7.4	0.0	3.7	0.0	0.0	11.1	11.1	9.7	27
3	96.4	0.0	0.0	3.6	0.0	0.0	3.6	3.6	22.5	28
4	83.3	5.6	0.0	5.6	5.6	0.0	16.7	16.7	29.1	18
6	90.3	0.0	0.0	0.0	9.7	0.0	9.7	9.7	22.7	31
7	96.9	0.0	0.0	0.0	3.1	0.0	3.1	3.1	32.1	32
8	96.8	3.2	0.0	0.0	0.0	0.0	3.2	3.2	26.6	31
9	96.6	0.0	3.4	0.0	0.0	0.0	3.4	3.4	31.6	29
10	100.0	0.0	0.0	0.0	0.0	0.0	0.0	0.0	35.2	30
11	92.3	7.7	0.0	0.0	0.0	0.0	7.7	7.7	40.6	39
12	38.9	61.1	0.0	0.0	0.0	0.0	61.1	61.1	85.1	18
13	26.5	73.5	0.0	0.0	0.0	8.8	64.7	73.5	89	34
14	45.2	54.8	0.0	0.0	0.0	16.1	38.7	54.8	87.9	31
15	100.0	0.0	0.0	0.0	0.0	0.0	0.0	0.0	20.6	30


*% S, the percentage of genotyped individuals with two susceptible alleles; % R, the percentage of genotyped individuals with one or two resistance alleles of the specified type; % RR alleles, the percentage of genotyped individuals homozygous for a resistance allele; % R alleles, the percentage of genotyped individuals heterozygous for a resistance allele; Total % R alleles, the total percentage of genotyped individuals with one or two resistance alleles; % R individuals, the percentage of phenotyped individuals surviving glyphosate treatment at 1681 g ae ha^-1^; N_G_, the number of individuals genotyped in each population*.

**FIGURE 1:**
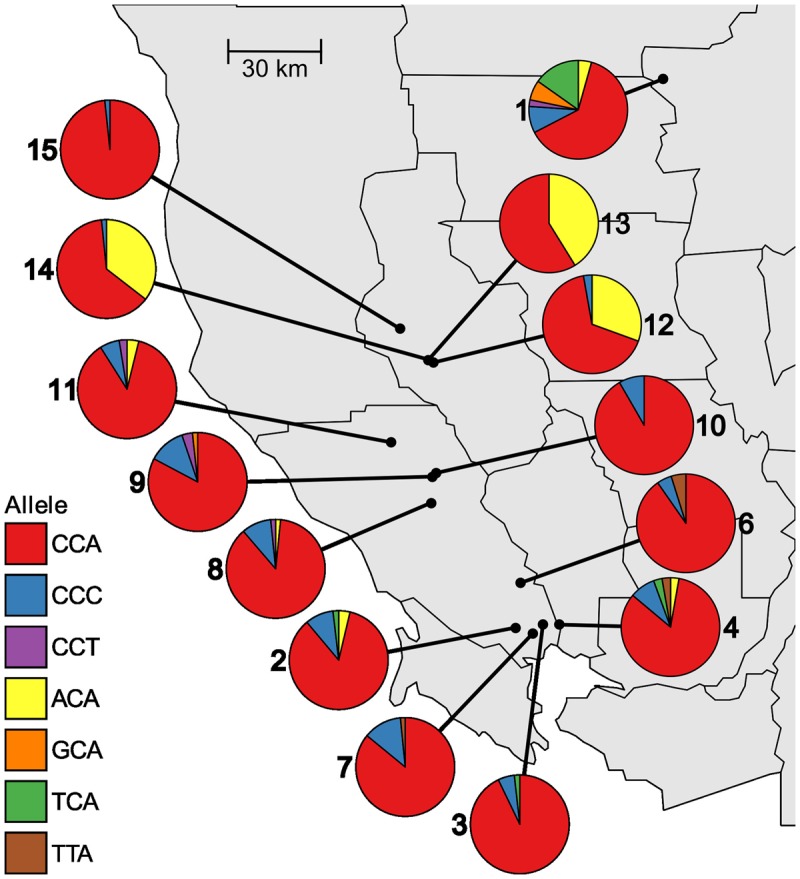
**Distribution and frequencies of *EPSPS* alleles at site 106 in 14 sampled populations of *Lolium perenne* ssp. *multiflorum* in northwest California**.

Within populations, the frequency of different alleles varies widely across the studied area. The existence of four separate alleles that confer resistance to glyphosate indicates multiple independent evolutionary origins of resistance. Of the four resistance alleles, P106T encoded by ACA is the most common and was found in 8 of the 14 populations (**Table 2**). P106T was detected at high frequencies (>50% of individuals) in Lake County populations 12, 13, and 14, which are located very near each other in the northern part of the sampled area (**Figure 1**). This suggests a common origin of the allele in this area, with subsequent spread to other nearby populations. The other three resistance alleles are found at lower frequencies in only a small number of populations each (**Table 2**), and are distributed mostly in the southern end of the studied area, and in population 1 (**Figure 1**). Population 1 is from an area that has had evolved glyphosate resistance for the longest period of time and contains three of the four different resistance alleles (**Table 2** and **Figure 1**). The three susceptible wild-type alleles, CCA, CCC, and CCT, all code for the same amino acid and would be expected to be selectively neutral. However, only the CCA version of P106 was found in all populations (**Table 2**).

For many populations, the frequency of individuals with glyphosate-resistant phenotypes correlates roughly with the frequency of individuals with resistance alleles (**Table 3**).

In all but one population, the frequency of resistant individuals is higher than the frequency of resistance alleles. In populations 10 and 15, no resistance alleles were detected despite the presence of 35.2 and 20.6% resistant individuals, respectively (**Table 3**), suggesting non-target-site-based mechanisms of resistance.

## Discussion

### Target-Site-Based Resistance

Resistance to glyphosate in northwest California populations of *L. perenne* ssp. *multiflorum* is commonly conferred by *EPSPS* target-site mutations at the site corresponding to codon 106, based on the results of this study. The four non-synonymous mutations identified in the sampled populations have been previously shown to confer glyphosate resistance in *L. perenne* ssp. *multiflorum* and ssp. *rigidum* (Wakelin and Preston, 2006; Jasieniuk et al., 2008; Kaundun et al., 2011; Sammons and Gaines, 2014). The presence of four separate resistance alleles indicates at least four separate evolutionary origins of target-site-based glyphosate resistance across the agricultural landscape. Moreover, the distribution of resistance alleles suggests that resistance either evolved independently in the southern and northern extremes of the studied area, and/or there has been long-distance gene flow between these areas through movement of resistant weed seed. Local spread of resistance through gene flow is also likely, as demonstrated by the shared presence of some resistance alleles in closely located populations.

Most resistance alleles detected can be explained by a single nucleotide substitution from a susceptible allele, except for the P106L allele encoded by TTA, which requires two nucleotide substitutions from CCA or three from CCC or CCT. It is also possible that the P106L allele is a result of a single nucleotide substitution from the P106S allele encoded by TCA. P106L and P106S do co-occur in one population. However, both alleles are relatively uncommon, making exact determinations of the origins of allele P106L difficult.

The number of individuals genotyped in this study does not allow detection of rare alleles (*p* < 0.05) within populations. Very large sample sizes are required to detect rare genetic variants and diminishing returns of increasing sample sizes makes detection of all rare alleles in a population impractical (Marshall and Brown, 1975). The threshold for the desired level of allele detection varies with the goal of the study, e.g., collection of rare adaptive genotypes for breeding or maintenance of representative germplasm for conservation (Crossa and Vencozsky, 2011). To be 95% confident in detecting at least one copy of all alleles with frequency *p* > 0.05, it is necessary to sample 59 unrelated gametes, or approximately 30 diploid outcrossing individuals, while only 15 individuals are required to detect all alleles with frequency *p* > 0.1 (Marshall and Brown, 1975). The goal of this study was to detect mutant alleles that confer glyphosate resistance in *L. perenne* populations. In orchard and vineyard populations with frequencies of resistant plants high enough to be of concern to weed management, the alleles conferring the resistance trait likely are also common. However, it is possible that populations, which currently are predominantly susceptible, contain rare resistance alleles that may increase in frequency in the future due to selection, and this study may not have detected those alleles.

### Non-Target-Site-Based Resistance

Most sampled populations contained a higher frequency of resistant individuals than resistant *EPSPS* alleles, indicating that non-target-site-based mechanisms may underlie resistance in some individuals. The differences in frequencies of resistant phenotypes and resistant genotypes may also be partially due to stochastic effects associated with the plants used for phenotyping versus those used for genotyping, or due to error in the estimation of allele frequencies from the relatively low number of plants genotyped. Both methodological issues could result in estimates of genotype frequencies deviating from their actual values in populations, and would have an equal probability of skewing estimates to be either higher or lower than the actual genotype frequencies. However, 13 of the 14 sampled populations have higher frequencies of resistant plants than resistance alleles (**Table 3**), strongly suggesting a biological cause rather than stochastic effects. Non-target-site mechanisms of glyphosate resistance have been identified in *L. perenne* populations from other agricultural areas in the United States as well as worldwide (Perez-Jones et al., 2007; Preston et al., 2009; Salas et al., 2012, 2015). In addition to the differences in frequencies of resistant phenotypes and genotypes, further support for the presence of non-target-site-based resistance in this study comes from populations 10 and 15, in which no resistance alleles were detected despite the populations containing 35.2% and 20.6% resistant individuals, respectively (**Table 3**). It is highly unlikely that resistant *EPSPS* alleles could be present at those frequencies in a population without being detected in 30 genotyped individuals. It is far more likely that these populations contain individuals that are resistant to glyphosate through a non-target-site-based mechanism. Individuals with a non-target-site-based mechanism of resistance would be phenotyped as resistant but genotyped as containing only P106 alleles.

Altered translocation of glyphosate away from growing tissue and overexpression of the EPSPS enzyme through gene duplication have both been identified as the mechanisms underlying resistance to glyphosate in populations of *L. perenne* in other agricultural regions (Wakelin et al., 2004; Perez-Jones et al., 2007; Salas et al., 2012, 2015). The genetic basis of altered translocation is not currently known, thus adaptive genetic variation associated with the mechanism could not be analyzed here. *EPSPS* gene duplication was not detected in California populations (Putta and Jugulam, 2015, personal communication). It is also possible that individual plants have multiple mechanisms of resistance, containing both target-site-based and non-target-site-based mechanisms of resistance. Little is known about whether separate mechanisms of resistance to the same herbicide may confer an increased level of resistance, or if a fitness cost to one or both herbicides may affect the frequencies of one or both mechanisms in future generations. Future physiological and genetic studies will assess the relative importance of target-site and non-target-site-based mechanisms of resistance to glyphosate in *L. perenne* populations of California.

The presence of both target-site and non-target-site resistance in a region indicates that the evolution of herbicide resistance can be quite complex across an agricultural landscape. A similar pattern of multiple target-site alleles with additional non-target-site-based mechanisms conferring herbicide resistance was observed for *Alopecurus myosuroides* and *L. perenne* ssp. *rigidum* evolving resistance to ACCase-inhibitors (Delye et al., 2010b; Malone et al., 2014), and indicates this may be common in weeds across agricultural landscapes. Resistance traits in populations located near each other may have separate or shared evolutionary histories through novel mutation of resistance alleles or spread through gene flow. Single populations may contain heterogeneous mixtures of individuals with distinct resistance traits. This is especially important to recognize considering that due to experimental limitations, studies of herbicide resistance in weeds often investigate only a small number of populations or only a few individual plant lines from a larger number of populations, and may result in studies concluding resistance is more uniform than it is in reality.

## Conclusion

Resistance to glyphosate has evolved repeatedly in populations of *L. perenne* ssp. *multiflorum* across the agricultural landscape of northwest California. Four distinct alleles at codon site 106 of the *EPSPS* gene confer target-site resistance to glyphosate. The distribution of *EPSPS* alleles among and within populations reveals a complex evolutionary history of the resistance trait, with multiple independent mutation events together with local spread of resistance through gene flow. Recently, non-target-site-based resistance is becoming evident in some populations within the region further complicating identification of the evolutionary origins and processes underlying resistance. It is clear, however, that long-term successful management of glyphosate-resistant *L. perenne* will require the adoption of strategies to manage currently resistant populations while also reducing the selection pressure for future evolution of glyphosate resistance.

## Author Contributions

EK and MJ have made substantial, direct and intellectual contributions to the work, and approved it for publication.

## Conflict of Interest Statement

The authors declare that the research was conducted in the absence of any commercial or financial relationships that could be construed as a potential conflict of interest.
